# The impact of uncertainty in society on the use of traditional, complementary and alternative medicine: a comparative study on visits to alternative/traditional/folk health care practitioners

**DOI:** 10.1186/s12906-019-2662-x

**Published:** 2019-09-09

**Authors:** Jimpei Misawa, Rie Ichikawa, Akiko Shibuya, Yukihiro Maeda, Ichiro Arai, Teruyoshi Hishiki, Yoshiaki Kondo

**Affiliations:** 1 Department of Health Care Services Management, Nihon University School of Medicine, 30-1, Oyaguchikami-cho, Itabashi, Tokyo, 173-8610 Japan; 2 Department of Pediatrics and Child Health, Nihon University School of Medicine, 30-1, Oyaguchikami-cho, Itabashi, Tokyo, 173-8610 Japan; 3 Department of Kampo Medicine, Nihon Pharmaceutical University, 10281, Komuro, Ina, Kita-adachi, Saitama, 362-0806 Japan; 4 Department of Information Science, Faculty of Science, Toho University, 2-2-1, Miyama, Funabashi, Chiba, 274-8510 Japan

**Keywords:** Traditional, complementary and alternative medicine, International social survey Programme, Uncertainty in society, Job insecurity

## Abstract

**Background:**

While traditional, complementary and alternative medicine (TCAM) is gaining increased interest worldwide, the structural factors associated with the usage of TCAM at the social level have not been sufficiently explored. We aim to understand the social structure of uncertainty in society that affects the TCAM usage for men and women.

**Methods:**

We studied 32 countries using data from the International Social Survey Programme and the World Bank. In this study, we defined TCAM usage as visits to an alternative/traditional/folk health care practitioner during the past 12 months. We performed a correlation analysis and used a generalized linear model .

**Results:**

The prevalence of TCAM usage in terms of visits to practitioners was 26.1% globally, while usage varied across the 32 countries. Generalized linear models showed that unemployment rate was associated with the prevalence of TCAM usage in terms of visits to practitioners.

**Conclusions:**

At the social-structural level TCAM usage involving visits to practitioners was related to job insecurity. Job insecurity led to a decrease in TCAM usage regarding visits to practitioners. These findings suggest that it is necessary to consider the social-structural factors of uncertainty in society when designing health policies related to TCAM.

**Electronic supplementary material:**

The online version of this article (10.1186/s12906-019-2662-x) contains supplementary material, which is available to authorized users.

## Background

Complementary and alternative medicine (CAM) is defined as “a broad domain of resources that encompasses health systems, modalities, and practices and their accompanying theories and beliefs, other than those intrinsic to the dominant health system of a particular society or culture in a given historical period. CAM includes such resources perceived by their users as associated with positive health outcomes. Boundaries within CAM and between the CAM domain and the domain of the dominant system are not always sharp or fixed” [1]. In recent years, interest in CAM has increased [2, 3]. Moreover, traditional medicine (TM) has always maintained its popularity worldwide [4]. The combination of TM and CAM is called traditional, complementary and alternative medicine (TCAM) [5, 6]. The importance of studies on the use of TCAM has been emphasised [6]. Existing literature has reported that the prevalence of TCAM usage among adults in some developed countries ranges between 5 and 76% [3, 7]. In the United States, the proportion of people using some form of TCAM was 32.3% in 2002, 35.5% in 2007, and 33.2% in 2012 (based on age-adjusted data) [8]. The prevalence of TCAM usage in EU countries also varied widely, between 0.3 and 86% [9], as well as in Asian countries. According to a national telephone survey conducted in 2001 for the general population in Japan, the proportion of TCAM usage was 76% [10], and in South Korea, 71.3% of people reported having had at least one TCAM therapy during the previous 12 months [11]. According to a national health interview survey of the general population in Taiwan in the same year, the proportion of TCAM usage was 31.8% among men and 22.4% among women [12]. These results show that the prevalence of TCAM usage varies greatly across countries and that there is a high level of interest in TCAM the world over.

Despite the high interest in TCAM usage overall, previous studies have focused on individual factors and not studied the factors affecting TCAM usage from a macro perspective so far. Among the individual factors, biological determinants have been reported to be related to TCAM usage. Various types of TCAM among various countries are used by women [13–16], middle-aged people [16–19], and people with health issues [16, 20–22]. Furthermore, TCAM usage was found to be related to individual psychological determinants including hope [23], health anxiety [24], and belief in the efficacy of TCAM [25, 26]. In addition to these biological and psychological determinants, TCAM usage is also associated with sociological determinants such as culture and customs that surround the healthcare domain. Educational level [16, 19, 27], income [21, 28, 29], and residential areas [21, 30, 31] were found to be associated with TCAM usage.

In the countries where TCAM usage was studied, the social structure was generally related to these individual factors. Indeed, it has been suggested that individual psycho-social factors were affected by the social structure [32]. Furthermore, Gale (2014) has indicated that TCAM was an important social phenomenon [33]. Moreover, it was noted that there was a need to clarify the factors underlying TCAM usage from the perspective of social structures [23]. Additionally, it was indicated that what is considered complementary or alternative care may have country-level variations [34]. Thus, it is necessary to examine the social-structural factors at the cross-country level. However, previous research that elucidated pertinent factors of TCAM usage has largely ignored the effect of social structures on TCAM usage and has focused only on individual factors. Since the global prevalence of TCAM usage varies across countries, it is necessary to examine these rates of prevalence to understand the associated social-structural factors underlying TCAM usage at the social level.

Various social-structural problems exist at the social level that need to be solved. Modern society is filled with uncertainty and risk [35]. For example, uncertain contexts existing within a society may include social inequality, ageing population, and job insecurity. However, with the weakening of various norms, people are forced to manage various uncertainties and risks by themselves [35]. In such uncertain social contexts, people experience various anxieties in their lives [36]. With regard to the relationship between health and uncertainty in society, previous researches have reported that uncertain social contexts, such as social inequality, economic recession, and ageing population are harmful to health [37–39]. Furthermore, previous studies have shown that various anxieties in life, such as job insecurity, adversely affect the health of individuals experiencing them [40, 41]. Based on these facts, we speculate that people in uncertain social contexts will become sufficiently interested in their own health to cope with uncertainties and risks. TCAM usage can be considered one of the health behaviors. The increased popularity of self-management of health has resulted in an increased interest in TCAM usage [2]. Furthermore, Frass et al. referenced reports on TCAM usage and interests in the US, Europe, and Asia-Pacific countries, and argued that the interest in TCAM has grown over the past decade. Moreover, the general public’s attitude toward TCAM is largely positive [3]. Considering this, in uncertain contexts, people will use TCAM due to their growing interest in maintaining their own health. Accordingly, we hypothesize that the social structure of uncertainty in society is positively related to TCAM usage. Furthermore, with regard to interests in health, previous studies have indicated that women are more interested in health and are more likely to search for health-related information than men [42, 43]. Indeed, as for the relationship between gender and TCAM usage, studies in each country have indicated that women use TCAM more than men [13–16]. Additionally, studies have shown that men and women have different coping styles when exposed to stressful life events, such as unemployment [44, 45]. Thus, we also suppose that the impact of uncertainty in society on TCAM usage varies between genders.

We aim to understand the social structure of uncertainty in society that affects the TCAM usage for men and women. We identify how this social structure of uncertainty contributes to variations in the prevalence of TCAM usage. Clarifying the factors involved in using TCAM from a social-structural point of view, and not from the perspective of individual factors, can help develop social policy about TCAM in the future.

## Methods

### Study design and dataset

We conducted an ecological study using the country as the study unit to better understand global TCAM utilization rates. We also conducted a secondary analysis using an archived dataset from the International Social Survey Programme (ISSP): Health and Health Care [46] to determine the prevalence of TCAM usage. The survey was conducted from February 2011 through April 2013 in the countries listed in Table 1. The sample size was 55,081. The largest sample size was China (*n* = 5620) and the smallest was the United Kingdom (*n* = 936). When the gross sample size was calculated as the denominator, the response rate was 48.0% in the total sample. The highest was in South Africa (85.8%) and the lowest was in Italy (23.4%). Furthermore, Table 1 briefly demonstrates the survey methods, sampling methods, presence of weighting, and age structure for each country (See codebooks of ISSP [46] for more information on these). In every country, the subjects were randomly sampled from the electoral list or the list of national registrations. The survey methods involved mainly face-to-face interviews and postal surveys. The youngest participant was 15-years-old, while the oldest was 102-years-old. The age structure varied among countries. The survey data weighted to correct for bias included 21 out of 32 countries. However, as there is no weight available for the international comparison on the ISSP among countries, we calculated the age-standardized prevalence of TCAM usage in order to take into consideration the age structure of each country.

**Table 1:** Social survey summary of each of the 32 target countries

Countries	Date of Collection	Gross sample size^a^	Sample size	Response rate^b^	Min age	Max age	Mean age	Analyzed sample^c^	Survey methods	Sampling methods	Weight
Australia	May 2012 - Aug 2012	6250	1946	31.1	18	97	55.1	1830	Postal survey (self-completion)	Random sampling	Yes
Belgium	Apr 2011 - Dec 2012	8821	3083	35.0	17	95	49.7	2850	Face-to-face interview (CAPI) and mail survey	Stratified two-stage random sampling and Simple random sampling	Yes
Bulgaria	Aug 2011 - Sep 2011	2275	1003	44.1	18	90	51.9	969	Face-to-face (PAPI) interview	Three stage random sampling	Yes
Chile	Nov 2011 - Dec 2011	1872	1559	83.3	18	99	46.5	1436	Face-to-face interview	Three stage random sampling	Yes
China	Jun 2011 - Nov 2011	7800	5620	72.1	17	97	47.8	5558	Face-to-face interview	Multi-stage stratified sampling	Yes
Taiwan	Jul 2011 - Apr 2012	4424	2199	49.7	18	93	46.8	2196	Face-to-face interview (pencil and paper & CAPI)	Three-stage stratified PPS sampling	Yes
Croatia	May 2011 - Jun 2011	2576	1210	47.0	18	87	45.6	1132	Face to face interview	Multistage sampling	No
Czech Republic	Feb 2012 - Mar 2012	3230	1804	55.9	18	92	47.4	1696	Face-to-face interview	Four stage stratified probability sampling	Yes
Denmark	Jan 2013 - Mar 2013	2500	1388	55.5	18	102	46.3	1364	Web based mail survey (self-completion)	Simple random sampling	No
Finland	Aug 2011 - Dec 2011	2500	1340	53.6	15	75	46.2	1309	Postal, paper and pencil or internet survey (self-completion)	Systematic sampling	Yes
France	Mar 2011 - Sep 2011	10,000	3319	33.2	18	95	52.1	3025	Postal survey	Random equal probability sampling	Yes
Germany	Apr 2012 - Sep 2012	5103	1681	32.9	18	95	49.5	1632	Self-completion questionnaire (CASI) and face-to-face interview (CAPI)	Two stage random sampling	No
Israel	Nov 2011 - Apr 2012	1594	1220	76.5	18	92	45.8	1084	Face to face interview	Four stage stratified sampling	No
Italy	Oct 2012 - Feb 2013	5062	1186	23.4	16	92	50.7	1074	Self-completion questionnaire (paper and pencil) was delivered by interviewer and returned by mail	Four stage stratified sampling	Yes
Japan	Nov 2011 - Dec 2011	1800	1306	72.6	16	100	50.5	1287	Self-completion	Two-stage stratified random sampling	No
South Korea	Jun 2011 - Aug 2011	2500	1535	61.4	18	94	46.0	1535	Face-to-face interview	Multi-stage area probability sampling	No
Lithuania	Nov 2011 - Dec 2011	3313	1187	35.8	18	87	47.7	1099	Face to face interview/paper and pencil interview (PAPI), with visuals	Multistage stratified sampling	Yes
Netherlands	Mar 2011 - Dec 2011	4500	1472	32.7	17	97	54.0	1406	Postal survey	Simple random sampling	Yes
Norway	Mar 2012 - Apr 2012	3800	1834	48.3	18	77	48.3	1789	Postal and web survey	Random sampling	No
Philippines	Sep 2011 - Sep 2011	3206	1200	37.4	18	86	42.9	1167	Face-to-face interviews with visuals	Multi-stage Probability Sampling	Yes
Poland	Apr 2013 - Apr 2013	2640	1115	42.2	18	88	47.8	1081	CAPI	Multi-stage area probability sampling	Yes
Portugal	Nov 2012 - Apr 2013	2256	1022	45.3	18	93	51.6	968	CAPI	Four stage area sampling	Yes
Russia	Dec 2011 - Dec 2011	3170	1511	47.7	18	96	48.1	1467	Face-to-face interviews	Four stage stratified probability sampling	Yes
Slovakia	Oct 2012 - Dec 2012	2544	1128	44.3	18	92	51.9	1111	CAPI with visuals	Two stage sampling	Yes
Slovenia	Mar 2011 - Jun 2011	1800	1082	60.1	18	99	48.6	1062	Personal interviews (pencil and paper with visuals)	Two-stage stratified random sampling	No
South Africa	Sep 2011 - Oct 2011	3500	3004	85.8	16	95	40.6	2757	Face-to-face interview	Multi-stage stratified sampling	Yes
Spain	May 2012 - Jul 2012	4000	2712	67.8	18	97	49.2	2622	Face-to-face interview	Two phased, stratified by clusters. Proportional random sampling	Yes
Sweden	Feb 2011 - May 2011	1966	1158	58.9	18	80	50.0	1087	Postal survey	A representative sample of the Swedish population	No
Switzerland	Mar 2011 - Nov 2011	2409	1212	50.3	19	98	48.9	1192	CAPI	Simple random sampling	No
Turkey	Nov 2011 - Jan 2012	3150	1559	49.5	17	92	42.1	1398	Face-to-face interview with visuals	Multi-stage area sampling	No
UK	Jul 2011 - Nov 2011	2260	936	41.4	17	97	49.7	897	Face-to-face interview and postal survey (self-completion)	Clustered random sampling	Yes
US	Mar 2012 - Sep 2012	2044	1550	75.8	20	89	50.0	1512	Face-to-face interview with CAPI	Multi-stage area probability sample	Yes
Total	Mar 2011 - Mar 2013	114,865	55,081	48.0	15	102	48.3	52,592			

*CAPI* Computer-Assisted Personal Interview, *PAPI* Paper-And-Pencil Interview, *PPS* Probability Proportional to the Size

^a^Total number of starting target subjects

^b^The proportion of number of respondents to gross sample size (%)

**Fig. 1:**
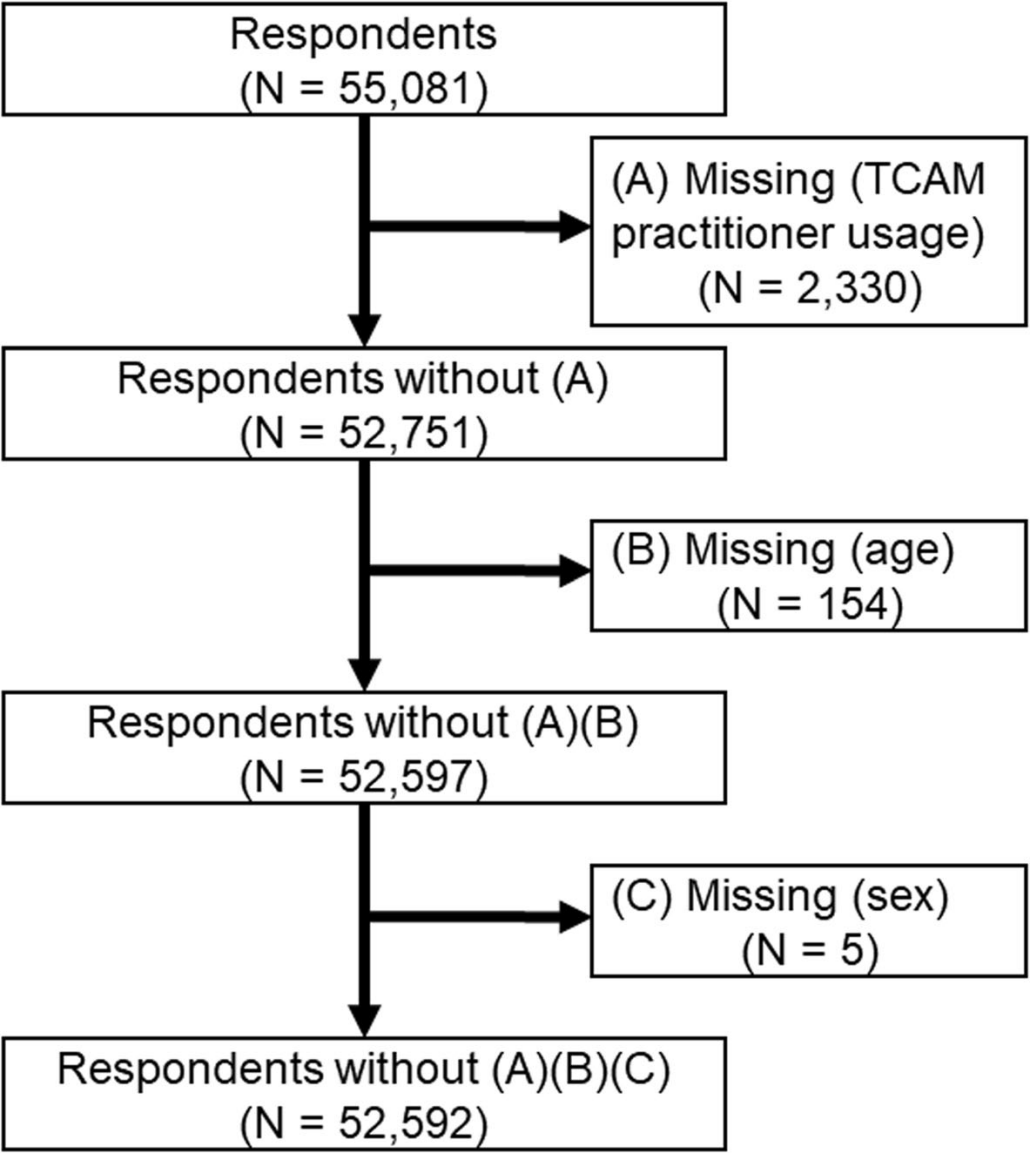
The flowchart of the process of TCAM practitioner usage, age and gender excluded. Samples with missing information on TCAM practitioner usage, age and sex variables were excluded. Finally, 52,592 samples were analysed

^c^A flow chart demonstrates the process through which the TCAM practitioner usage, age, and sex were excluded in Fig. 1. The excluding process for each country has been shown in Additional file 1: Table S1

Participants in the ISSP survey were asked the following question: “During the past 12 months, how often did you visit or were visited by an alternative/traditional/folk health care practitioner?” They had to select one of the following options: never, seldom, sometimes, often, or very often. We grouped these responses into two values of “uses TCAM” (containing “seldom” to “very often” responses) and “does not use TCAM” (containing “never” responses) in order to calculate the prevalence of TCAM usage in each of the 32 countries. Therefore, the definition of TCAM usage in this study implies visits to an alternative/traditional/folk health care practitioner during the past 12 months; this is referred to as “TCAM practitioner usage” in this study. The data that was analyzed included only those participants for whom there were no missing values for TCAM practitioner usage, sex and age (*n* = 52,592). In Fig. 1, a flow chart demonstrates the process through which TCAM practitioner usage, age, and sex were excluded (Fig. 1). The excluding process for each country has been shown in Additional file 1: Table S1.

We evaluated the social-structural factors of uncertainty in society for each country from a three-fold perspective: inequality, occupation, and population. We employed the Gini index as the inequality perspective, the country’s unemployment rate as the occupation perspective, and the ageing rate as the population perspective. The Gini index is an indicator of income inequality within the country. As the value gets closer to 100, inequality becomes greater. We employed the index because countries with high levels of income inequality are considered uncertain societies. Unemployment rate is an indicator of job insecurity within the country. We relied on the unemployment rate since countries with high levels of job insecurity are considered uncertain societies. Ageing rate is an indicator of population structure within the country. We relied on the ageing rate as the indicator of uncertainty in society since a high proportion of elderly people is related to a small labor force and declining birthrates. The Gini index, unemployment rate, and ageing rate were adopted from the World Bank dataset [47] for the same 31 countries (since data for Taiwan was missing) as those in the ISSP survey data. We used the average values between 2011 and 2013 for these variables. When a country did not have data for this period, we chose the year that is closer to 2011 to 2013 as another year (see Table 2 for details).

**Table 2:** Statistics of social structural indicators of the 32 target countries

Countries	Gini index^a^	Unemployment rate^a^	Ageing rate^a^	GDP per capita^a^
Australia	34.7^d^	5.3^b^	14.0^b^	65,863.0^b^
Belgium	27.8^b^	7.7^b^	17.6^b^	46,342.0^b^
Bulgaria	35.6^b^	12.2^b^	18.9^b^	7622.2^b^
Chile	47.5 ^h^	6.5^b^	9.7^b^	15,359.7^b^
China	42.2^g^	4.4^b^	8.8^b^	6349.8^b^
Taiwan	NA	NA	NA	NA
Croatia	32.4^b^	15.6^b^	18.0^b^	13,783.3^b^
Czech Republic	30.7 ^h^	6.9^b^	16.4^b^	20,454.4^b^
Denmark	27.9^b^	7.4^b^	17.5^b^	60,484.1^b^
Finland	27.3^b^	7.9^b^	18.4^b^	49,281.5^b^
France	32.9^b^	9.8^b^	17.5^b^	42,400.8^b^
Germany	30.7 ^h^	5.5^b^	20.8^b^	45,802.2^b^
Israel	41.4^f^	6.7^b^	10.7^b^	34,169.0^b^
Italy	34.8^b^	10.4^b^	21.2^b^	36,173.0^b^
Japan	32.1^c^	4.3^b^	23.9^b^	45,742.0^b^
South Korea	31.6^f^	3.2^b^	11.5^b^	24,776.2^b^
Lithuania	34.3^b^	13.5^b^	17.8^b^	14,803.9^b^
Netherlands	27.8^b^	6.0^b^	16.4^b^	51,529.9^b^
Norway	25.8^b^	3.2^b^	15.4^b^	101,812.9^b^
Philippines	42.2^f^	7.0^b^	4.3^b^	2564.9^b^
Poland	32.6^b^	10.0^b^	14.2^b^	13,604.8^b^
Portugal	36.2^b^	14.8^b^	19.5^b^	21,797.4^b^
Russia	40.5^b^	5.8^b^	13.2^b^	15,264.3^b^
Slovakia	26.9^b^	13.9^b^	13.0^b^	17,884.5^b^
Slovenia	25.6^b^	9.0^b^	17.1^b^	23,625.2^b^
South Africa	63.4^e^	24.6^b^	4.8^b^	7425.6^b^
Spain	35.8^f^	24.1^b^	17.8^b^	29,870.3^b^
Sweden	27.2^b^	8.0^b^	18.8^b^	59,003.5^b^
Switzerland	31.9^b^	4.2^b^	17.3^b^	85,688.8^b^
Turkey	40.1^b^	8.5^b^	7.4^b^	11,868.1^b^
United Kingdom	32.9^b^	7.8^b^	17.2^b^	41,975.7^b^
United States	41.0^g^	8.1^b^	13.6^b^	51,342.6^b^
Mean	34.6	9.1	15.2	34,344.1
Standard Deviation	7.6	5.1	4.6	23,662.0
Maximum	63.4	24.6	23.9	101,812.9
Minimum	25.6	3.2	4.3	2564.9

^a^From the World Bank dataset

^b^Data (average) for the period from 2011 to 2013

^c^Data for 2008

^d^Data for 2010

^e^Data for 2011

^f^Data for 2012

^g^Data for 2013

^h^Data (average) of 2011 and 2013

We also employed Gross Domestic Product (GDP) per capita as a control variable. In this study, since we conducted an ecological macro analysis at the national level, it is necessary to adjust for the wealth of the country. GDP per capita was also adopted from the World Bank dataset [47]. As for the variable, we adopted the average value between 2011 and 2013, as mentioned above.

### Statistical analysis

We first calculated the age-standardized prevalence of TCAM practitioner usage among the 32 target countries for the entire sample and then by gender. Next, we conducted a correlation analysis (Spearman’s rho: *r*_*s*_) that calculated simple correlation coefficients among the Gini index, unemployment rate, ageing rate, GDP per capita, and the prevalence of TCAM practitioner usage. We conducted a generalized linear model (Gamma distribution with log link function) with the prevalence of TCAM practitioner usage as the outcome variable for the entire sample and by gender as the outcome variables were continuous and skewed [48]. First, model 1, wherein the Gini index, unemployment rate, and ageing rate was incorporated, was analyzed respectively. Next, model 2, wherein all variables were incorporated, was analyzed. The GDP per capita was controlled in both models. *P*-values of < 0.05 were considered statistically significant.

## Results

**Fig. 2:**
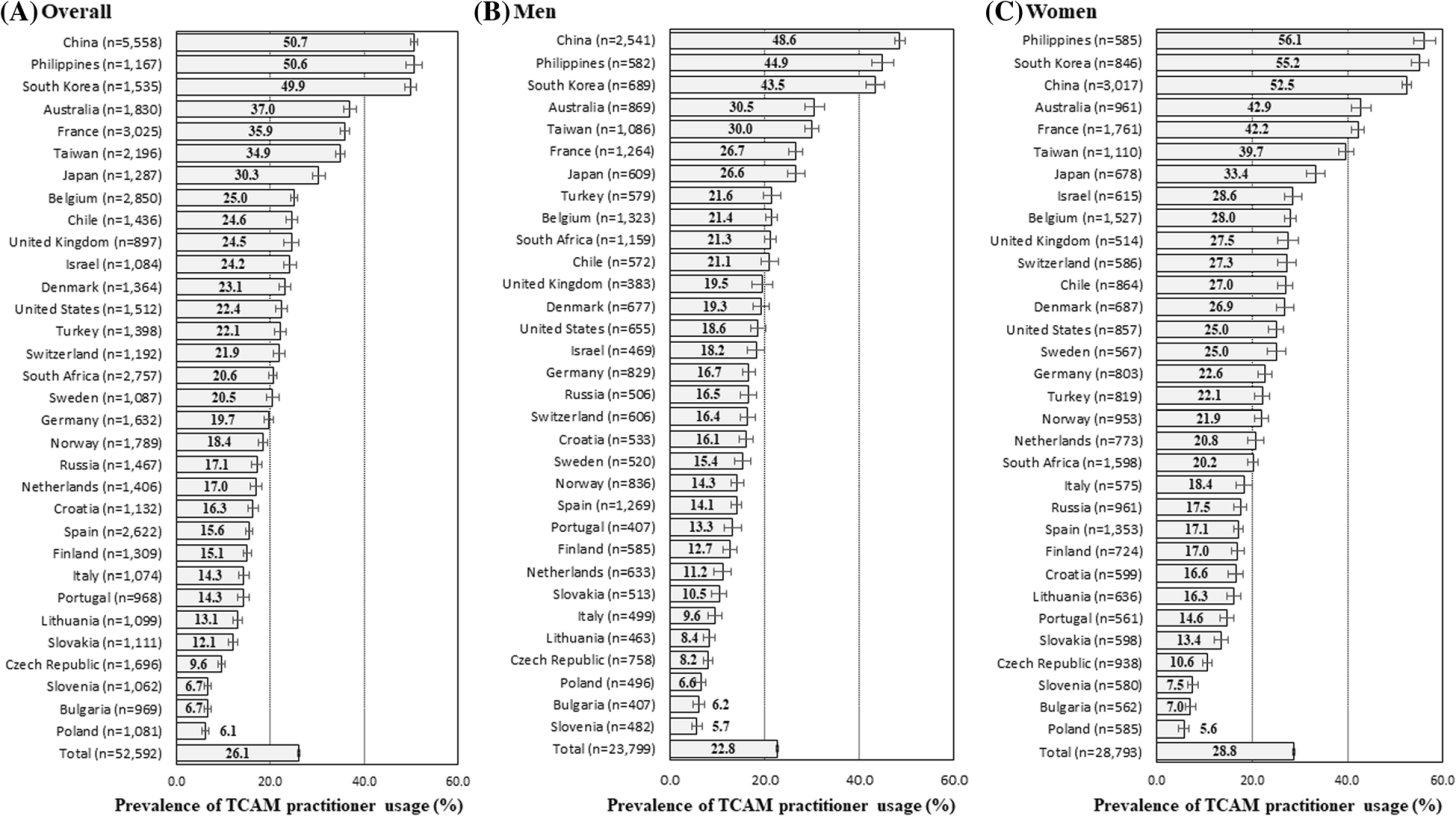
The prevalence of TCAM practitioner usage among 32 countries. Prevalence of TCAM practitioner usage was stratified by country for (**a**) the Entire Sample, (**b**) Men, and (**c**) Women. Values in parentheses represent the sample size of each country

Of the 52,592 participants available in the ISSP survey data, 26.1% had used TCAM practitioner in the past 12 months (Fig. 2). The prevalence of TCAM practitioner usage in the past 12 months was 22.8% among men and 28.8% among women. For the entire sample, the highest and lowest TCAM practitioner usage prevalence rates were 50.7% in China and 6.1% in Poland. For men, the highest and lowest TCAM practitioner usage prevalence rates were 48.6% in China and 5.7% in Slovenia. For women, the highest and lowest TCAM practitioner usage prevalence rates were 56.1% in Philippines and 5.6% in Poland.

Table 2 shows statistics of the social-structural indicators, with the mean Gini index at 34.6. The mean unemployment rate and ageing rate were 9.1 and 15.2%, respectively. The mean GDP per capita applied as the control variable was 34,344.1.

Table 3 shows the results of the correlation coefficients between social-structural factors of uncertainty and the prevalence of TCAM practitioner usage for the entire sample and by gender. For the entire sample, we found a statistically significant negative correlation of the prevalence of TCAM practitioner usage with n the unemployment rate (*r*_*s*_ = − 0.549, *P* = 0.001) and the ageing rate (*r*_*s*_ = − 0.373, *P* = 0.039). However, a statistically significant correlation was not found between the Gini index and the prevalence of TCAM practitioner usage (*r*_*s*_ = 0.314, *P* = 0.085). For men, we found a statistically significant positive correlation of the prevalence of TCAM practitioner usage with the Gini index (*r*_*s*_ = 0.400, *P* = 0.026), and a statistically significant negative correlation of the prevalence of TCAM practitioner usage with the unemployment rate (*r*_*s*_ = − 0.435, *P* = 0.015) and the ageing rate (*r*_*s*_ = − 0.420, *P* = 0.019). For women, we found a statistically significant negative correlation of the prevalence of TCAM practitioner usage with the unemployment rate (*r*_*s*_ = − 0.610, *P* < 0.001). However, a statistically significant correlation was not found among the Gini index (*r*_*s*_ = 0.237, *P* = 0.199), the ageing rate (*r*_*s*_ = − 0.299, *P* = 0.102), and the prevalence of TCAM practitioner usage. GDP per capita was not correlated with the prevalence of TCAM practitioner usage for the entire sample, among men, or among women. Among the social-structural indicators of uncertainty, the Gini index was negatively correlated with the ageing rate (*r*_*s*_ = − 0.417, *P* = 0.020).

**Table 3:** Associations with the prevalence of TCAM practitioner usage for total sample and by gender

	Prevalence of TCAM practitioner usage	Prevalence of TCAM practitioner usage for men	Prevalence of TCAM practitioner usage for women	Gini index	Unemployment rate	Ageing rate	GDP per capita
Prevalence of TCAM practitioner usage for total sample	1.000	0.963	0.977	0.314	−0.549	− 0.373	0.165
		(< 0.001)	(< 0.001)	(0.085)	(0.001)	(0.039)	(0.374)
Prevalence of TCAM practitioner usage for men		1.000	0.901	0.400	−0.435	−0.420	0.011
			(< 0.001)	(0.026)	(0.015)	(0.019)	(0.954)
Prevalence of TCAM practitioner usage for women			1.000	0.237	−0.610	− 0.299	0.269
				(0.199)	(< 0.001)	(0.102)	(0.143)
Gini index				1.000	0.159	−0.417	−0.557
					(0.394)	(0.020)	(0.001)
Unemployment rate					1.000	0.211	−0.422
						(0.254)	(0.018)
Ageing rate						1.000	0.376
							(0.037)
GDP per capita							1.000

Values in parentheses represent *P*-values

Table 4 shows the results of the generalized linear model as controlled by GDP per capita for the total sample and by gender. For the entire sample, the Gini index was not related to the prevalence of TCAM practitioner usage in model 1.1 (B = 0.029, *P* = 0.064). However, for the entire sample, the unemployment rate (B = − 0.041, *P* = 0.031) and ageing rate (B = − 0.059, *P* = 0.009) were related to the prevalence of TCAM practitioner usage in model 1.2 and 1.3, respectively. In model 2, for the entire sample, the unemployment rate was related to the prevalence of TCAM practitioner usage (B = − 0.044, *P* = 0.027). For men, the ageing rate was related to the prevalence of TCAM practitioner usage in model 1.3 (B = − 0.061, *P* = 0.009). However, for men, the Gini index (B = 0.032, *P* = 0.050) and the unemployment rate (B = − 0.038, *P* = 0.054) were not related to the prevalence of TCAM practitioner usage in model 1.1 and 1.2, respectively. In model 2, for men, the unemployment rate was related to the prevalence of TCAM practitioner usage (B = − 0.042, *P* = 0.039). For women, the Gini index was not related to the prevalence of TCAM practitioner usage in model 1.1 (B = 0.0.28, *P* = 0.079). However, for women, the unemployment rate (B = − 0.043, *P* = 0.025) and the ageing rate (B = − 0.059, *P* = 0.010) were related to the prevalence of TCAM practitioner usage in model 1.2 and 1.3, respectively. In model 2, for women, the unemployment rate was related to the prevalence of TCAM practitioner usage (B = − 0.045, *P* = 0.026).

**Table 4:** Generalized linear model with the prevalence of TCAM practitioner usage controlled by GDP per capita (*N* = 31)

Overall	Model 1.1			Model 1.2			Model 1.3			Model 2		
	B	SE	*P* value	B	SE	*P* value	B	SE	*P* value	B	SE	*P* value
Intercept	1.890	0.635	0.006^**^	3.550	0.268	< 0.001***	3.717	0.306	< 0.001***	2.985	0.818	0.001**
Gini index	0.029	0.015	0.064							0.022	0.016	0.175
Unemployment rate				−0.041	0.018	0.031*				−0.044	0.019	0.027*
Aging rate							−0.059	0.021	0.009**	−0.031	0.025	0.221
Deviance	7.7			7.1			6.5			5.3		
AIC	237.3			234.8			232.0			229.3		
Men												
Intercept	1.723	0.645	0.012**	3.445	0.279	< 0.001***	3.665	0.317	< 0.001***	2.843	0.843	0.002**
Gini index	0.032	0.015	0.050							0.024	0.016	0.157
Unemployment rate				−0.038	0.019	0.054				−0.042	0.019	0.039*
Aging rate							−0.061	0.022	0.009**	−0.033	0.026	0.207
Deviance	7.8			7.6			6.7			5.4		
AIC	227.6			226.6			222.4			220.0		
Women												
Intercept	1.999	0.642	0.004**	3.620	0.269	< 0.001***	3.759	0.307	< 0.001***	3.104	0.827	< 0.001***
Gini index	0.028	0.015	0.079							0.021	0.016	0.211
Unemployment rate				−0.043	0.018	0.025*				−0.045	0.019	0.026*
Aging rate							−0.059	0.021	0.010*	−0.031	0.025	0.230
Deviance	8.0			7.2			6.9			5.5		
AIC	245.3			242.0			240.1			237.6		

*B* Coefficient

*SE* Standard error

*AIC* Akaike’s Information Criterion

***: *p* < 0.001; **: *p* < 0.01; *: *p* < 0.05

## Discussion

In this study, we found that the prevalence of TCAM practitioner usage varied across the 32 countries. We also found that the unemployment rate, as uncertainty in society, was associated with the prevalence of TCAM practitioner usage.

Employing an international social survey conducted between 2011 and 2013, this study was able to determine the prevalence of TCAM practitioner usage across 32 countries. We found that the prevalence of TCAM practitioner usage was high in Asian countries. This might be owing to popular traditional medicine practices in these countries, such as traditional Chinese medicine and Kampo medicine [10, 12, 20, 23]. Overall, the prevalence of TCAM usage in the present study tended to be lower than that found in previous research [7, 8, 10], with an average of 26.1%. Most previous surveys have included TCAM products and practitioners [8, 10, 23]. The ISSP survey, however, investigated the visits to an alternative/traditional/folk health care practitioner during the past 12 months as TCAM practitioner usage. Thus, the prevalence of TCAM usage in this study tended to be lower than that found in previous research. However, compared to a previous review on the prevalence of TCAM usage regarding visits to TCAM practitioners [7], the prevalence of TCAM usage in this study was not very low. Thus, we believe that, in terms of the prevalence of TCAM practitioner usage, the findings of this study are reasonable. We also found that the TCAM practitioner usage prevalence rate among women (28.8%) was higher than that among men (22.8%). These findings support previous studies showing the differences between men and women at an individual level [13–16]. Thus, we believe that the results at the social-structural level in this study are valid.

Previous research on the prevalence of and factors affecting TCAM usage has not focused on an international comparison. Rather, it has examined the impact of individual determinants on TCAM usage. Since questionnaires to determine whether participants use TCAM vary depending on the study context, it was difficult to accurately compare the prevalence of TCAM usage between countries. This current survey, however, found that the prevalence of TCAM practitioner usage varied greatly among the 32 target countries. Analyzing the international comparison survey data precisely showed the variations in the prevalence of TCAM usage among the target countries. The findings in this study show that the difference between the maximum and minimum prevalence rates among 32 countries was about 44 points for the entire sample. The difference was quite large among men and women. The difference among women was more prominent. This suggests that the utilization of TCAM varies widely from county to country, and that the unique characteristics of a country may be related to the prevalence of TCAM usage. The large difference between the maximum and minimum prevalence rates among women in comparison to those among men suggests that women might use TCAM considerably depending on the social context of the country.

We analyzed the relationship between the social structure of uncertainty — inequality, occupation, and population perspectives — and the prevalence of TCAM practitioner usage. According to the results of the generalized linear models with all variables on uncertainty in society, we found a negative relationship between the unemployment rate (i.e., the occupation perspective) and the prevalence of TCAM practitioner usage in the entire sample, for both men and women. This result suggests that uncertainty in employment might lead to people refraining from using TCAM as a consumption behavior. This may be because people consider TCAM as a kind of luxury, and so they cannot afford to use TCAM. In particular, as the TCAM usage in this study was defined as visits to an alternative/traditional/folk health care practitioner, rather than TCAM as products that people can easily obtain, TCAM usage might be a luxury for people in uncertain contexts with job insecurity. Therefore, people might not use TCAM to steadily cope with uncertainty in a society with job insecurity.

Furthermore, differences in the cost of using TCAM by country may be relevant to people for considering TCAM as a luxury. For example, in the United States, TCAM visits to practitioners of services such as acupuncture and Ayurveda are higher in out-of-pocket costs than TCAM products such as non-vitamin, non-mineral, and natural products [49]. Conversely, in Japan, where TCAM has been used traditionally, there is no significant difference between the costs of visits to TCAM practitioners such as acupuncture specialists and TCAM products such as Kampo; this is because individuals in Japan are reimbursed by health insurance for using these services and products [10]. In other words, in countries where the cost of TCAM is high, high unemployment rate may be related to lesser usage of TCAM. Based on these points, although this paper focused only on the social-structural factors of uncertainty in society, it will be necessary in future studies to explore how TCAM usage is institutionalized in the healthcare systems of each country, as well as to examine whether people can easily use TCAM in terms of cost.

The fact that people do not use TCAM may also indicate that people sacrifice their own health to cope with uncertainty in society. We hypothesized that the social structure of uncertainty in society is positively related to TCAM usage, and speculated that people will use TCAM more to maintain their health in an uncertain society. However, the findings did not confirm this hypothesis. Therefore, we argue that people rely on steady coping behaviors to deal with uncertainty in a society with job insecurity, instead of using TCAM (as they may consider it to be a luxury). Regarding the relationship between job insecurity and health status, previous researchers have indicated that people with job insecurity are not healthy [40, 41, 50]. Thus, based on these findings, it appears that not using TCAM may lead to a deterioration of the health status of socially disadvantaged people in an uncertain society with job insecurity. Additionally, we found that only the unemployment rate was related to TCAM practitioner usage, and not the Gini index or ageing rate. This may be because people consider job insecurity as an easily-understood uncertain social context, rather than income inequality and the ageing population. It would be reasonable to consider that uncertainty in terms of employment may lead to people refraining from using TCAM as it is seen as a luxury, given that the middle-aged population, who are active workers, tended to use TCAM frequently [16, 51]. As this study has shown that an increase in the unemployment rate is negatively related to TCAM usage, especially visits to alternative/traditional/folk health care practitioners, it would be appropriate to take economic measures for promoting TCAM usage. As a previous study has shown that the health of the population suffers after economic recession [38], economic measures may be effective for health policy regarding TCAM usage.

Furthermore, and interestingly, it appears that the pathway of the impact of unemployment rate on TCAM practitioner usage differs slightly between men and women. For women, the effect of unemployment rate on TCAM practitioner usage was found to be consistent from model 1.2 (i.e., the model adopting only the unemployment rate controlled by GDP per capita) to model 2 (i.e., the model adopting all variables). However, for men, although unemployment rate had no significant effect on TCAM practitioner usage in model 1.2, the effect of unemployment rate does appear in model 2. Therefore, this finding partly supports the hypotheses that the impact of an uncertain society on TCAM usage varies between genders. The findings suggest that, for men, it would be reasonable to consider that unemployment rate influences the prevalence of TCAM usage in relation to other social-structural factors of uncertainty in society. Certain economic measures for men against unemployment are necessary, considering the influences of income inequalities and ageing population.

Regarding the social-structural variables of uncertainty in society other than unemployment rate, we were unable to determine the effect of the Gini index (i.e., income inequality) on TCAM usage in the form of visits to alternative/traditional/folk health care practitioners. Previous research has shown that people living in countries with large income inequalities have lower population health than those in countries with low income inequalities [37]. Furthermore, a previous study reported a significant relationship between TCAM usage, excellent health, and health improvement [52]. With respect to the pathways from income inequality to health, studies have shown that psychosocial factors, such as social capital and trust, mediate the relationship [53, 54]. Thus, psychosocial factors could mediate the relationship between income inequality and TCAM usage. However, as this is beyond the scope of this study, and considering the impact of income inequalities on TCAM usage, it will be necessary to consider psychosocial factors as well in future study.

Additionally, regarding the ageing rate, we found that although ageing rate had a significantly negative effect on TCAM practitioner usage in model 1.3 (i.e., the model adopting only the ageing rate controlled by GDP per capita), the effect of ageing rate disappeared in the model that adopted all variables (model 2). This finding may also be involved in the influence of other social-structural factors of uncertainty in society. The results of the correlation analysis showed that the ageing rate was moderately correlated with the Gini index and GDP per capita. Previous studies have reported that population ageing was associated with income inequalities [55]. Thus, population ageing is strongly associated with social structural elements such as income inequalities. Therefore, we believe that population ageing is not associated with TCAM usage directly when other social-structural factors of uncertainty in society are involved. In that sense, even while considering the other social structural factors,, job insecurity was shown to be more important factors underlying TCAM usage at a national level.

The study showed that CAM usage is related to the occupational perspective (especially job insecurity) in social structure. For future research and policy recommendations, it is necessary to examine the occupational perspectives in social structure while considering the macro-level factors underlying TCAM usage. Since many countries do not have any health policy that takes TCAM into account [56], it will be necessary to take job insecurity in the social structure into account while designing future health policies related to TCAM.

There are some limitations in the present study. First, we analyzed the relationship between uncertainty in society and the prevalence of TCAM usage at the macro level to understand its global prevalence among 32 countries. However, examining the effect of social structure of uncertainty on TCAM usage at the individual level will be useful too. By understanding the factors underlying TCAM usage and by taking into consideration the influence of the individual and macro levels, it will be possible to clarify the more essential factors underlying the use of TCAM. Moreover, we suggested the effects of economic measures when arguing from an employment perspective. Previous research has shown that economic measures at the national level have an effect on population health [57]. However, another study has shown that economic incentives are not always related to people’s motivation to improve their own health [58]. Thus, there may be limits to the economic measures at the national level. Based on these points, it will be necessary to survey the trends of economic measures in each country and to explore how such measures relate to the relationship between unemployment rate and TCAM usage. In addition, the finding of the present study suggested that not using TCAM may lead to deterioration of health status in an uncertain society. However, in western countries, not all people opt for TCAM usage even if they have a health complaint and can afford it [59]. Thus, considering the relationship between TCAM usage, health, and economic conditions, the impact of uncertainty in society on TCAM usage should be elucidated. We also analyzed the cross-sectional relationship among factors in the study using the ISSP data. As response rate tends to be low in countries where postal surveys were conducted, the data may be biased. However, we calculated the age-standardized prevalence of TCAM practitioner usage to take into account the bias, such as the age structure, of each country. Furthermore, because social structures of uncertainty change over time, it will be necessary to re-examine the longitudinal effects of social structure of uncertainty on TCAM usage. Since research on the relationship between social structure of uncertainty and TCAM usage is scarce, solving these limitations in future studies will serve to clarify the relationship. Finally, the definition of TCAM in the present study was limited to visits to alternative/traditional/folk health care practitioners. Generally, the definition of TCAM does not only include these practices, but also products such as supplements. The most commonly used TCAM products is supplements [8, 10, 23]. Furthermore, the definitions of TCAM in a local context are crucial for comparisons. Moreover, the definition of TCAM in the present study includes both complementary and traditional therapies. Thus, although the impact of uncertainty in society on the use of TM and CAM could be different, the difference might be overlooked. This difference should be considered when examining the effect of social structure of uncertainty on TCAM usage not only at the social level but also at the individual level. Therefore, in future studies, it will be important to examine the relationship between uncertain social structure and the use of TCAM including supplements, while considering not only the definition of TCAM in the local contexts but also the difference between TM and CAM.

## Conclusions

At the social level, the prevalence of TCAM usage regarding visits to practitioners among the 32 target countries showed great variations. The usage of TCAM regarding visits to practitioners was shown to be related to the social-structural factors of uncertainty. Job insecurity decreased TCAM usage regarding visits to practitioners. These findings suggest that it is necessary to consider the social-structural factors of uncertainty in society when designing health policies related to TCAM.

## Additional file

Additional file 1: **Table S1.** The process of TCAM practitioner usage, age and gender exclusion in 32 countries. The process in which TCAM practitioner usage, age and sex were excluded in each country is shown. (DOCX 16 kb)

## Data Availability

The datasets generated and/or analyzed during the current study are available in the gesis repository (10.4232/1.12252) and the World Bank Open Data (http://data.worldbank.org/).

## Abbreviations

CAM: Complementary and Alternative Medicine
GDP: Gross Domestic Product
ISSP: International Social Survey Programme
*r*_*s*_: Spearman’s rho
TCAM: Traditional, Complementary and Alternative Medicine
TM: Traditional Medicine
