# Supplementary material for: The impact of uncertainty in society on the use of traditional, complementary and alternative medicine: a comparative study on visits to alternative/traditional/folk health care practitioners
Source: BMC Complement Altern Med. 2019 Sep 9;19:251. doi: 10.1186/s12906-019-2662-x (PMC6734350; doi:10.1186/s12906-019-2662-x)
Supplement: Supplementary file 1 — Table S1. The process of TCAM practitioner usage, age and gender exclusion in 32 countries. The process in which TCAM practitioner usage, age and sex were excluded in each country is shown. (DOCX 16 kb) [file 12906_2019_2662_MOESM1_ESM.docx]

Additional file 1

**Table S1 The process of TCAM practitioner usage, age and gender exclusion in 32 countries**

| **Countries** | **Sample size** |  | **Missing (TCAM practitioner usage)** |  | **Missing (Age)** |  | **Missing (Sex)** |  | **Analyzed sample** |
| --- | --- | --- | --- | --- | --- | --- | --- | --- | --- |
| Australia | 1946 | 🡪 | 81 | 🡪 | 35 | 🡪 | 0 | 🡪 | 1830 |
| Belgium | 3083 | 🡪 | 215 | 🡪 | 17 | 🡪 | 1 | 🡪 | 2850 |
| Bulgaria | 1003 | 🡪 | 34 | 🡪 | 0 | 🡪 | 0 | 🡪 | 969 |
| Chile | 1559 | 🡪 | 123 | 🡪 | 0 | 🡪 | 0 | 🡪 | 1436 |
| China | 5620 | 🡪 | 62 | 🡪 | 0 | 🡪 | 0 | 🡪 | 5558 |
| Taiwan | 2199 | 🡪 | 3 | 🡪 | 0 | 🡪 | 0 | 🡪 | 2196 |
| Croatia | 1210 | 🡪 | 65 | 🡪 | 13 | 🡪 | 0 | 🡪 | 1132 |
| Czech Republic | 1804 | 🡪 | 108 | 🡪 | 0 | 🡪 | 0 | 🡪 | 1696 |
| Denmark | 1388 | 🡪 | 24 | 🡪 | 0 | 🡪 | 0 | 🡪 | 1364 |
| Finland | 1340 | 🡪 | 31 | 🡪 | 0 | 🡪 | 0 | 🡪 | 1309 |
| France | 3319 | 🡪 | 294 | 🡪 | 0 | 🡪 | 0 | 🡪 | 3025 |
| Germany | 1681 | 🡪 | 47 | 🡪 | 2 | 🡪 | 0 | 🡪 | 1632 |
| Israel | 1220 | 🡪 | 127 | 🡪 | 9 | 🡪 | 0 | 🡪 | 1084 |
| Italy | 1186 | 🡪 | 75 | 🡪 | 33 | 🡪 | 4 | 🡪 | 1074 |
| Japan | 1306 | 🡪 | 19 | 🡪 | 0 | 🡪 | 0 | 🡪 | 1287 |
| South Korea | 1535 | 🡪 | 0 | 🡪 | 0 | 🡪 | 0 | 🡪 | 1535 |
| Lithuania | 1187 | 🡪 | 82 | 🡪 | 6 | 🡪 | 0 | 🡪 | 1099 |
| Netherlands | 1472 | 🡪 | 66 | 🡪 | 0 | 🡪 | 0 | 🡪 | 1406 |
| Norway | 1834 | 🡪 | 45 | 🡪 | 0 | 🡪 | 0 | 🡪 | 1789 |
| Philippines | 1200 | 🡪 | 33 | 🡪 | 0 | 🡪 | 0 | 🡪 | 1167 |
| Poland | 1115 | 🡪 | 34 | 🡪 | 0 | 🡪 | 0 | 🡪 | 1081 |
| Portugal | 1022 | 🡪 | 51 | 🡪 | 3 | 🡪 | 0 | 🡪 | 968 |
| Russia | 1511 | 🡪 | 44 | 🡪 | 0 | 🡪 | 0 | 🡪 | 1467 |
| Slovakia | 1128 | 🡪 | 17 | 🡪 | 0 | 🡪 | 0 | 🡪 | 1111 |
| Slovenia | 1082 | 🡪 | 20 | 🡪 | 0 | 🡪 | 0 | 🡪 | 1062 |
| South Africa | 3004 | 🡪 | 244 | 🡪 | 3 | 🡪 | 0 | 🡪 | 2757 |
| Spain | 2712 | 🡪 | 90 | 🡪 | 0 | 🡪 | 0 | 🡪 | 2622 |
| Sweden | 1158 | 🡪 | 71 | 🡪 | 0 | 🡪 | 0 | 🡪 | 1087 |
| Switzerland | 1212 | 🡪 | 20 | 🡪 | 0 | 🡪 | 0 | 🡪 | 1192 |
| Turkey | 1559 | 🡪 | 149 | 🡪 | 12 | 🡪 | 0 | 🡪 | 1398 |
| UK | 936 | 🡪 | 39 | 🡪 | 0 | 🡪 | 0 | 🡪 | 897 |
| US | 1550 | 🡪 | 17 | 🡪 | 21 | 🡪 | 0 | 🡪 | 1512 |
| Total | 55081 | 🡪 | 2330 | 🡪 | 154 | 🡪 | 5 | 🡪 | 52592 |
